# The Modified Femoral Neck-Shaft Angle: Age- and Sex-Dependent Reference Values and Reliability Analysis

**DOI:** 10.1155/2016/8645027

**Published:** 2016-12-14

**Authors:** Christoph Kolja Boese, Michael Frink, Janine Jostmeier, Stefan Haneder, Jens Dargel, Peer Eysel, Philipp Lechler

**Affiliations:** ^1^Department of Orthopedic and Trauma Surgery, University Hospital of Cologne, Cologne, Germany; ^2^Center of Orthopedic and Trauma Surgery, University of Giessen and Marburg, Marburg, Germany; ^3^Institute of Diagnostic and Interventional Radiology, University Hospital of Cologne, Cologne, Germany

## Abstract

*Background*. The femoral neck-shaft angle (NSA) is of high importance for the diagnostics and treatment of various conditions of the hip. However, rotational effects limit its precision and applicability using plain radiographs. This study introduces a novel method to measure the femoral NSA: the modified NSA (mNSA), possibly being less susceptible against rotational effects compared to the conventional NSA.* Patients and Methods*. The method of measurement is described and its applicability was tested in 400 pelvis computed tomography scans (800 hips). Age- and gender-dependent reference values are given and intra- and interrater reliability are analyzed.* Results*. The mean age of all 400 patients (800 hips) was 54.32 years (18–100, SD 22.05 years). The mean mNSA was 147.0° and the 95% confidence interval was 146.7°–147.4°. Differences of the mNSA between sexes, age groups, and sides were nonsignificant. The absolute difference between NSA and mNSA was 16.3° (range 3–31°; SD 4.4°); the correlation was high (0.738; *p* < 0.001). Overall, the intra- and interrater reliability were excellent for the mNSA.* Interpretation*. We introduced a novel concept for the analysis of the neck-shaft angle. The high reliability of the measurement has been proven and its robustness to hip rotation was demonstrated.

## 1. Background

The femoral neck-shaft angle (NSA) is one of the most frequently applied measures of hip anatomy [1]. It is essential in the diagnosis of various pathological conditions of the hip and femur including developmental dysplasia of the hip (DDH) [2, 3], cerebral palsy (CP) in children and adults with consecutive pathologies of the hip [4], femoroacetabular impingement (FAI), Perthes disease and femoral head necrosis (FHN) [5], epiphysiolysis capitis femoris (ECF), osteogenesis imperfecta (OI), and proximal femoral fractures [6]. In particular the planning of operative interventions including osteotomies, hip replacement surgery, and internal fixation of fractures requires adequate preoperative and postoperative measurements of hip parameters.

**Figure 1:**
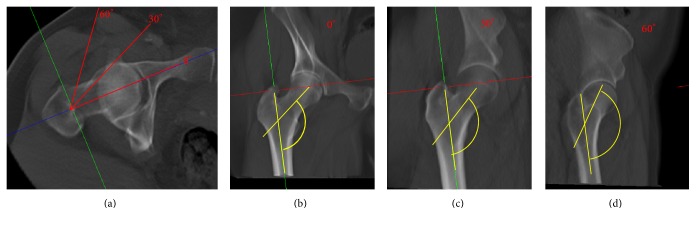
Effect of hip rotation on the projected neck-shaft angle (NSA). The axial view of the femur in a CT reconstruction of a right hip is shown (a). Three coronal reconstructions are projected. Internal hip rotation of 0°, 30°, and 60° is demonstrated as shown in the axial view. The NSA is included and the overestimation of the projected NSA due to the rotation is visualized.

To correctly assess the NSA, highly standardized anteroposterior radiographs are mandatory to avoid projectional errors of the projected NSA caused by hip rotation (Figure 1) [7–9]. Accounting for the suspected antetorsion of the femoral neck, 15 to 20° of internal rotation of the feet is recommended for standardized anteroposterior radiographs of the pelvis and hip. However, in patients with contractures (i.e., osteoarthritis, cerebral palsy) and those following musculoskeletal trauma, the overall mobility of the affected limb is usually severely restricted and the required internal rotation cannot be achieved [10].

The effect of internal and external hip rotation is an overestimation of the NSA (i.e., valgization), while flexion does lead to an underestimation (i.e., varization) [7, 8]. Figure 1 depicts the effect of hip rotation. Whereas complex biplanar radiographs were developed to account for these effects, their use is usually restricted to individual cases in paediatric orthopedics and associated with relatively high radiation exposure [11]. Additionally, the reliability of the measurement of the NSA has been challenged in recent publications and there exists no standard method of measurement in the current literature [8, 12–14]. Notably, a current systematic analysis of its application in healthy adults revealed a considerable heterogeneity in the distribution of the NSA [1].

This study aimed to present a novel method to measure the femoral NSA: the modified NSA (mNSA). We assumed that the modified NSA is less susceptible to rotational effects than the conventional NSA and is highly reproducible due to the exact definition of the anatomic landmarks. Thus, the mNSA might prove to be beneficial in the anatomic description of the hip for diagnostics and planning of surgery.

The method of measurement is described and the application was tested in 400 pelvis computed tomography scans (800 hips). Age- and gender-dependent reference values are given and intra- and interrater reliabilities were analyzed.

## 2. Patients and Methods

A previously described cohort of 400 patients undergoing whole-body trauma computed tomography scans was retrospectively included [15]. All CT scans were screened for inclusion and exclusion criteria. Here, the presence of fractures, deformities, implants, and immature skeleton served as exclusion criteria. Patients were stratified for further analysis into four groups consisting of 100 scans (200 hips) each: (A) male under 60 years of age, (B) male over 59 years of age, (C) female under 60 years of age, and (D) female over 59 years of age. A detailed description of the cohort has been published previously [15, 16].

Computed tomography scans were acquired using a Brilliance iCT 256 scanner (Philips Healthcare, Cleveland, OH, USA) and a standardized trauma-scan protocol was used. Radiographic data was deposited in a picture archiving and communication system (PACS) and analyzed using a PACS client (IMPAX EE; AGFA HealthCare GmbH, Bonn, Germany). Two independent observers performed radiologic measurements after a training-series of 10 cases. In 10% of all cases repeated measurements were performed by observer one and two 3 months after the first analysis, blinded to the previous results; intra- and interrater reliabilities were calculated.

### 2.1. Planes and Reconstructions for Measurements

To simulate nonstandardized radiographs of the pelvis, measurements were performed in the CT scout-view (Scout). To simulate semistandardized radiographs of the pelvis without optimal hip rotation, original axial CT images were reformatted in the anterior pelvic plane and a simulated radiograph was constructed (APP). Finally, a coronal reconstruction of each proximal femur in the plane of the femoral neck (FNP) was created, which eliminated rotational influences. All measurements followed a standardized protocol [15, 16]. The detailed methods of the reconstruction of the APP and FNP were described* previously* [15, 16].

### 2.2. The Modified Neck-Shaft Angle (mNSA)

**Figure 2:**
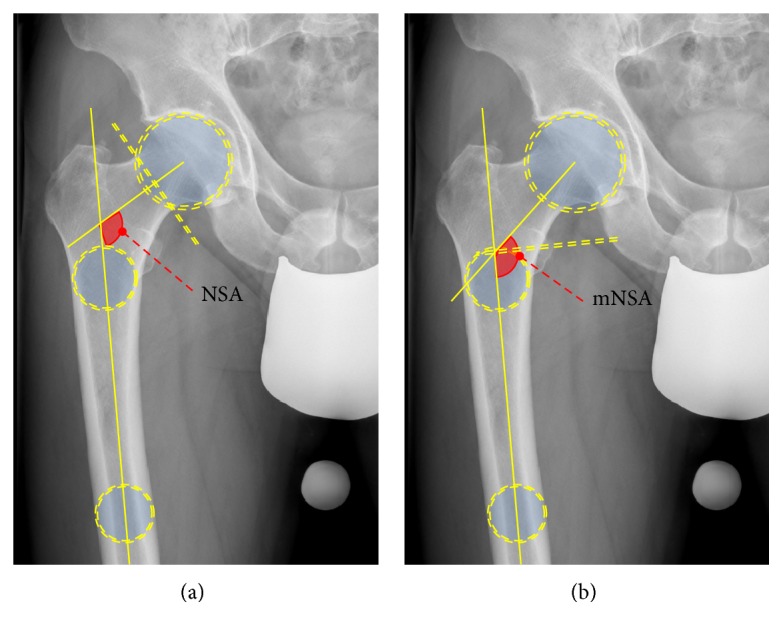
Schematic drawing of the measurement of the NSA (a) and mNSA (b). The NSA requires the definition of the centre of rotation and the waist of the femoral neck to define the femoral neck axis (FNA). Two circles in the femur are drawn to define the long femoral axis (FLA). The NSA is the angle between the FLA and FNA (a). The mNSA requires no FNA. A perpendicular to the FLA is drawn, cutting the apex of the minor trochanter. The cutting point of both axes is connected to the centre of rotation; the angle between this axis and the FLA is the mNSA.

The long axis of the femur (FLA) was defined by a line crossing the centre of two circles placed around the outer margins of the subtrochanteric femur at two distinct positions: the centre of the upper circle was positioned at the lower boundary of the minor trochanter and the lower circle was placed two centimeters below the first due to the end of the scan at this height. In radiographs and lower-reaching CT scans, the FLA is defined as depicted in Figure 2.

The modified femoral neck axis (mFNA) was defined as the line connecting the centre of rotation and the FLA at the height of the apex of the minor trochanter. Thus, a circle defined by three points around the margin of the femoral head was drawn, determining the centre of rotation. The cutting point with the FLA was found by drawing a perpendicular line from the FLA to the apex.

The modified NSA (mNSA) is the angle between the FLA and the modified FNA (mFNA). Figures 2(a) and 2(b) depict the NSA and the mNSA, respectively.

### 2.3. Statistics

For descriptive analysis, absolute mean values and ranges and standard deviations (SD) of the measured variables are reported. Variables were tested for normality using the Kolmogorov-Smirnoff test. Correlations of non-Gaussian distributed variables were described with the Spearman correlation coefficient (rho). Exploratory analysis was performed using the two-tailed Wilcoxon matched pair test for nonnormally distributed variables. For comparison of age distribution, the nonparametric Mann–Whitney *U* test was performed. Intra- and interrater reliabilities were evaluated using intraclass correlation coefficients (ICC). The level of significance was set at *p* < 0.05. IBM SPSS Statistics for Macintosh version 22.0 (IBM Corporation, Armonk, NY, USA) and Microsoft Excel 2008 for Mac version 12.3.6 (Microsoft Corporation, Redmond, USA) software were used.

## 3. Results 

### 3.1. Demographic Baseline Parameters

Per protocol, 200 CT scans for male patients and 200 CT scans for female patients were included. The mean age of all 400 patients (800 hip) was 54.32 years (18–100, SD 22.05 years). Mean age of females was 55.40 years (18–100; SD 22.41 years) and 53.24 years (18–89; SD 22.61 years) in males. Descriptive results for the measured mNSA in all planes are shown with means, ranges, and standard deviations for the complete cohort (Table 1), divided by sex (Table 2), by side (Table 3), by age (Table 4), and by the combination of age and sex (Table 5).

Overall, the mean mNSA was 147.0° and the 95% confidence interval was 146.7°–147.4°. The variance was 25.3° and the standard deviation was 5.0°.  Figure 4 shows a histogram of the frequencies of the mNSA in all 800 hips.

Differences of the mNSA between sexes were nonsignificant in all planes (Scout: *p* = 0.649; APP: *p* = 0.065; FNP: *p* = 0.468). The differences between age groups were significant (*p* < 0.001 in all planes). The spearman rho correlation coefficient showed only weak negative correlations between age and mNSA (Scout: rho = −0.351; APP: rho = −0.190; FNP: rho = −0.209; *p* < 0.001 for all).

Sides did not influence the mNSA (Scout: *p* = 0.696; APP: *p* = 0.890; FNP: *p* = 0.738).

The Kruskal-Wallis Test showed significant differences between the four age- and sex-stratified groups in all planes (*p* < 0.001) while pair-wise post hoc analysis showed nonsignificant results between old males and females as well as young males and females.

**Figure 3:**
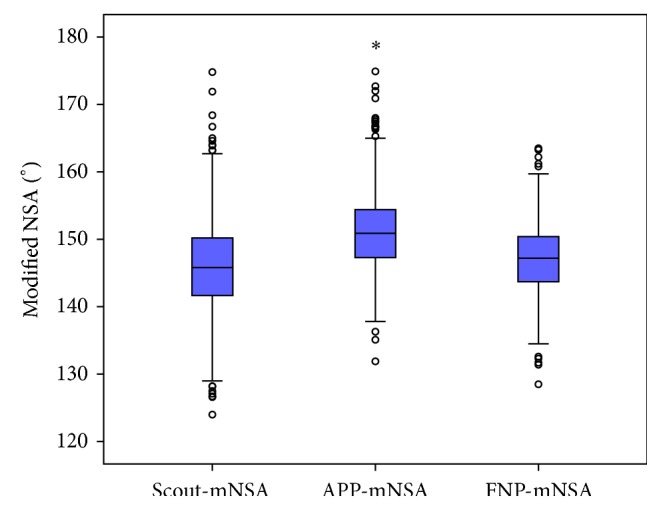
Box-Plot of the distribution of mNSA in 800 hips measured in three different planes: the scout-view, the anterior pelvic plane (APP), and the femoral neck plane (FNP). Asterisk (*∗*) is an extreme outlier. Circles are outliers of 1.5 times the IQR (interquartile range) and extreme outliers are beyond 3 times IQR.

**Figure 4:**
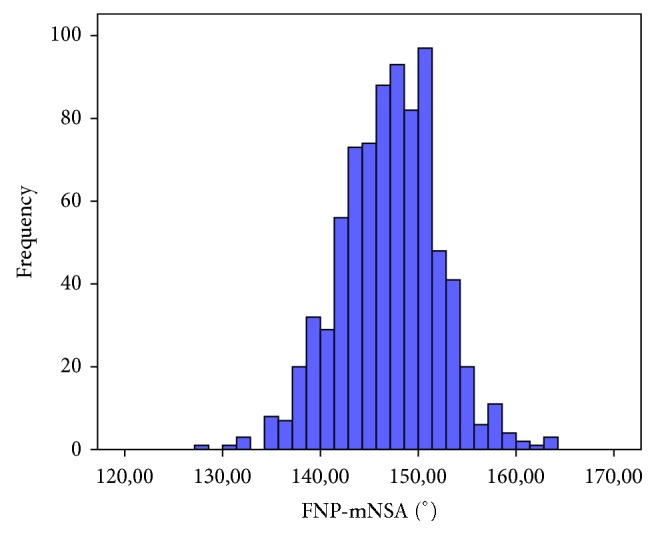
Histogram of the distribution of the mNSA in 800 hips in the femoral neck plane (FNP).

The comparison of the mNSA in the FNP and the scout or APP showed significant differences (both *p* < 0.001) (Box-Plot; Figure 3).

**Figure 5:**
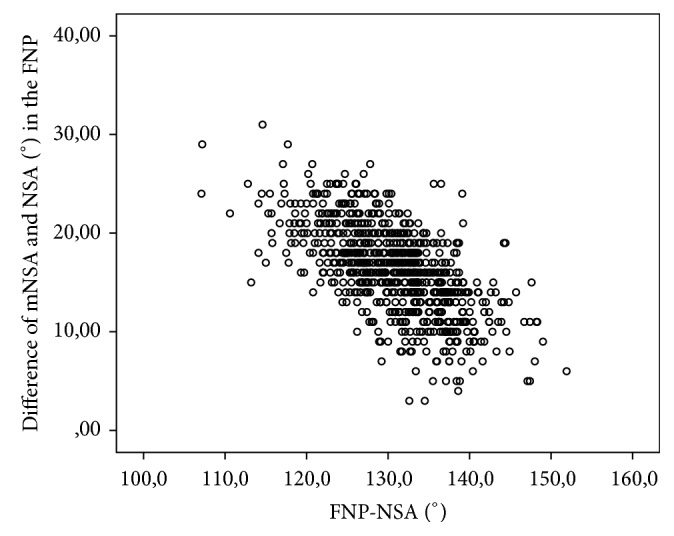
Scatterplot of the differences between the NSA and mNSA in 800 hips in the femoral neck plane (FNP).

**Figure 6:**
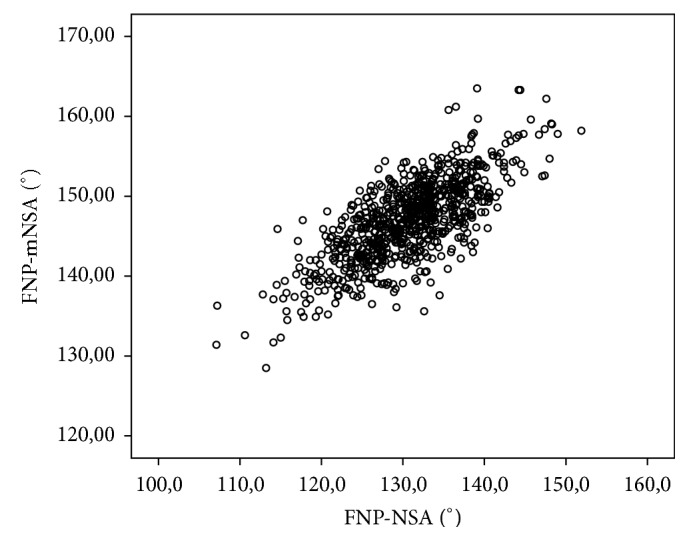
Scatterplot of the NSA and mNSA in 800 hips in the femoral neck plane (FNP).

The absolute difference between NSA and mNSA was 16.3° (Range 3–31°; SD 4.4°). There was a negative correlation between NSA and the absolute difference of mNSA and NSA (−0.633; *p* < 0.001), while mNSA and NSA correlated positively (0.738; *p* < 0.001). Scatterplots show their relationship in Figures 5 and 6.

ICCs are given in Tables 6 and 7 for repeated measurements of one and two independent observers. The ICC values for mNSA and NSA are given for each plane. Overall, the intra- and interrater reliability were excellent in all planes.

## 4. Discussion

The femoral neck-shaft angle is of highest clinical relevance for orthopedic and trauma surgeons and remains to be a subject of interest in current research [17, 18]. However, this specific parameter of proximal femoral anatomy has multiple limitations. First, the reproducibility has been questioned and various methods of measurement were described [1, 18]. While the method of Müller as presented by Boese et al. seems to allow for a sufficient reliability, a current systematic analysis of its application in the published literature emphasized the necessity to improve the method of its measurement [1]. Second, the NSA shows significant differences between ethnic groups, sexes, and age [19]. Of note, historical specimens showed significant differences compared to current populations [1, 19]. Third, the NSA is highly dependent from hip rotation and the projected NSA can differ significantly from the true (maximum) NSA (Figure 1) [7, 8, 15]. Even the application of highly standardized radiographs does not completely eliminate this effect [9]. Consequently, a number of recent studies aimed to provide novel reference values [1, 20].

The development of the modified NSA addresses all three limitations of the NSA.

First, the method of measurement is clearly defined. In particular, the problem to assess the femoral neck axis is avoided and a reproducible modified axis is used. The excellent intraclass correlation coefficients confirmed the high reproducibility of the method.

Second, the analyzed large cohort of 400 Caucasian patients without known pathologies to the hip covers both sexes and sides and the age distribution includes a wide range of adult patients. Thus, a representative sample for Caucasian patients was generated. Reference values for other ethnic groups can be provided easily by future studies. However, no sex-differences were found while age played a relevant role in the measured mNSA and NSA.

Third, we analyzed the effect of rotation by comparison of non- and semistandardized planes (Scout and APP) with the derotated plane (FNP).

As has been hypothesized, the mNSA shows higher values compared to the NSA with a narrower distribution. In particular, varus hips with low NSA values have higher values of differences from the mNSA compared to valgus NSA hips. Low-degree NSA hips are especially susceptible to rotation effects. Therefore, the robustness of the mNSA towards rotational effects counters this problem.

The authors are aware of several limitations of this study. First, the hypothetical superiority of the mNSA over the NSA in regard of applicability, reliability, and robustness towards hip rotation and flexion has not been proven in regard of an actual clinical setting. Still, the results of this investigation enable future clinical studies and supply researchers with highly reliable reference values. Second, the existing literature and clinical standards are based on the conventional determination of the NSA, which differs significantly from the mNSA. The discrepancy between both measures will limit its use in the clinical and scientific setting. While the benefits have been discussed, its superiority needs to be proven in the clinical application. Third, the examined cohort was retrospectively analyzed and the reference values are only valid in a comparable cohort of ethnic background and age. Thus, reference parameters should be generated for other groups as well.

In conclusion, this study introduced a novel concept for the assessment of the angulation of the proximal femur: the modified neck-shaft angle. In a large cohort, the high reliability of the measurements has been proven and the robustness to hip rotation was demonstrated. Furthermore, reference values for future studies were generated including side-, age- and sex-specific data.

## Figures and Tables

**Table 1:** Descriptive results for the modified neck-shaft angle (mNSA). Results of measurements in three planes of 800 hips are given. SD: standard deviation.

Variable	*n*	Mean	Range	SD
mNSA_Scout_	800	145.90	124.0–174.8	6.78
mNSA_APP_	800	151.00	131.9–178.8	5.90
mNSA_FNP_	800	147.04	128.5–163.5	5.03

**Table 2:** Descriptive results for the modified neck-shaft angle (mNSA) by sex. Results of measurements in three planes of 800 hips are given. SD: standard deviation.

	Sex							
	Female				Male			
	Mean	Minimum	Maximum	SD	Mean	Minimum	Maximum	SD
mNSA_Scout_	145.8	124.0	168.4	6.4	146.0	126.7	174.8	7.2
mNSA_APP_	150.5	137.8	168.0	5.5	151.5	131.9	178.8	6.3
mNSA_FNP_	147.08	131.40	162.20	5.01	147.00	128.50	163.50	5.05

**Table 3:** Descriptive results for the modified neck-shaft angle (mNSA) by side. Results of measurements in three planes of 800 hips are given. SD: standard deviation.

	Side							
	Right				Left			
	Mean	Minimum	Maximum	SD	Mean	Minimum	Maximum	SD
mNSA_Scout_	145.8	126.6	166.7	6.5	146.0	124.0	174.8	7.0
mNSA_APP_	150.9	131.9	178.8	5.7	151.1	135.1	174.9	6.1
mNSA_FNP_	146.99	131.40	163.50	4.82	147.10	128.50	162.20	5.24

**Table 4:** Descriptive results for the modified neck-shaft angle (mNSA) by age. Results of measurements in three planes of 800 hips are given. SD: standard deviation.

	Age group							
	Young (<60 years)				Old (≥60 years)			
	Mean	Minimum	Maximum	SD	Mean	Minimum	Maximum	SD
mNSA_Scout_	148.4	131.6	174.8	6.1	143.4	124.0	164.6	6.5
mNSA_APP_	152.2	138.8	174.9	5.5	149.8	131.9	178.8	6.0
mNSA_FNP_	148.22	136.60	163.50	4.57	145.86	128.50	162.20	5.20

**Table 5:** Descriptive results for the modified neck-shaft angle (mNSA) by group. Results of measurements in three planes of 800 hips are given. SD: standard deviation.

		Group			
		Female young	Female old	Male young	Male old
mNSA_Scout_	Mean	148.1	143.6	148.3	143.1
	Minimum	133.0	126.6	131.6	129.6
	Maximum	162.0	160.3	166.7	160.8
	SD	5.5	6.2	6.2	6.4

mNSA_APP_	Mean	151.7	149.5	152.2	150.2
	Minimum	138.8	137.9	139.5	131.9
	Maximum	167.5	165.3	167.5	178.8
	SD	5.3	5.5	5.4	6.4

mNSA_FNP_	Mean	148.12	145.86	147.99	145.98
	Minimum	137.60	131.40	136.60	135.60
	Maximum	157.80	156.90	163.50	157.70
	SD	4.27	4.88	5.02	4.67

**Table 6:** Intraclass correlation coefficient for repeated measurements of one observer.

Variable	Intraclass correlation	95% confidence interval	*p* value
mNSA_Scout_	0.983	0.973–0.989	<0.001
mNSA_APP_	0.986	0.978–0.978	<0.001
mNSA_FNP_	0.985	0.976–0.990	<0.001

**Table 7:** Intraclass correlation coefficient for repeated measurements of two independent observers.

Variable	Intraclass correlation	95% confidence interval	*p* value
mNSA_Scout_	0.949	0.920–0.967	<0.001
mNSA_APP_	0.801	0.690–0.873	<0.001
mNSA_FNP_	0.822	0.723–0.886	<0.001
